# Inducing sterile pyramidal neuronal death in mice to model distinct aspects of gray matter encephalitis

**DOI:** 10.1186/s40478-021-01214-6

**Published:** 2021-07-02

**Authors:** Justus B. H. Wilke, Martin Hindermann, Amir Moussavi, Umer Javed Butt, Rakshit Dadarwal, Stefan A. Berghoff, Aref Kalantari Sarcheshmeh, Anja Ronnenberg, Svenja Zihsler, Sahab Arinrad, Rüdiger Hardeland, Jan Seidel, Fred Lühder, Klaus-Armin Nave, Susann Boretius, Hannelore Ehrenreich

**Affiliations:** 1 Clinical Neuroscience, Max Planck Institute of Experimental Medicine, Hermann-Rein-Str.3, 37075 Göttingen, Germany; 2 Functional Imaging Laboratory, German Primate Center, Leibniz Institute for Primate Research, Kellnerweg 4, 37077 Göttingen, Germany; 3 Georg August University, Göttingen, Germany; 4 Department of Neurogenetics, Max Planck Institute of Experimental Medicine, Göttingen, Germany; 5 Johann Friedrich Blumenbach Institute of Zoology & Anthropology, University of Göttingen, Göttingen, Germany; 6 Institute for Neuroimmunology and Multiple Sclerosis Research, University Medical Center Göttingen, Göttingen, Germany

**Keywords:** Diphtheria toxin, Hippocampal learning and memory, (pre)frontal network dysfunction, Social cognition, Thermography, Magnetic resonance imaging

## Abstract

Up to one person in a population of 10,000 is diagnosed once in lifetime with an encephalitis, in 50–70% of unknown origin. Recognized causes amount to 20–50% viral infections. Approximately one third of affected subjects develops moderate and severe subsequent damage. Several neurotropic viruses can directly infect pyramidal neurons and induce neuronal death in cortex and hippocampus. The resulting encephalitic syndromes are frequently associated with cognitive deterioration and dementia, but involve numerous parallel and downstream cellular and molecular events that make the interpretation of direct consequences of sudden pyramidal neuronal loss difficult. This, however, would be pivotal for understanding how neuroinflammatory processes initiate the development of neurodegeneration, and thus for targeted prophylactic and therapeutic interventions. Here we utilized adult male NexCreERT2xRosa26-eGFP-DTA (= ‘DTA’) mice for the induction of a sterile encephalitis by diphtheria toxin-mediated ablation of cortical and hippocampal pyramidal neurons which also recruits immune cells into gray matter. We report multifaceted aftereffects of this defined process, including the expected pathology of classical hippocampal behaviors, evaluated in Morris water maze, but also of (pre)frontal circuit function, assessed by prepulse inhibition. Importantly, we modelled in encephalitis mice novel translationally relevant sequelae, namely altered social interaction/cognition, accompanied by compromised thermoreaction to social stimuli as convenient readout of parallel autonomic nervous system (dys)function. High resolution magnetic resonance imaging disclosed distinct abnormalities in brain dimensions, including cortical and hippocampal layering, as well as of cerebral blood flow and volume. Fluorescent tracer injection, immunohistochemistry and brain flow cytometry revealed persistent blood–brain-barrier perturbance and chronic brain inflammation. Surprisingly, blood flow cytometry showed no abnormalities in circulating major immune cell subsets and plasma high-mobility group box 1 (HMGB1) as proinflammatory marker remained unchanged. The present experimental work, analyzing multidimensional outcomes of direct pyramidal neuronal loss, will open new avenues for urgently needed encephalitis research.

**Supplementary Information:**

The online version contains supplementary material available at 10.1186/s40478-021-01214-6.

## Introduction

Encephalitis is best defined as inflammation of the brain parenchyma associated with variable neuropsychiatric dysfunction, ranging from mild to sometimes life-threatening severity. The underlying origins of encephalitis are multitude, and include infectious causes, comprising viral, bacterial, fungal, and parasitic agents, but also toxic, metabolic, (para)neoplastic and autoimmune or rarely post-immunization etiologies [29, 40, 42]. World travellers for example may be exposed to a variety of neurotropic pathogens leading to ‘exotic’ causes of central nervous system (CNS) infections [22]. Interestingly, increasing evidence points to genetic reasons of mild encephalitis which may often escape recognition until progressed age [17, 20, 37]. In about 50–70% of encephalitis cases, an etiologic agent is never identified, presumably due to the broad range of possible underlying origins and limitations in contemporary diagnostic testing, but likely also because of continuing pathogenetic dynamics in the absence of the formerly inducing agent [22, 29, 40, 42].

As recurrent common denominator in encephalitides and mediator of severe downstream sequelae, neuronal dysfunction or death are observed, associated with cognitive decline and dementia. Viral infections with neurotropism, particularly affecting neurons of cortex and hippocampus occur across many species. They include for instance human immunodeficiency, Theiler's murine encephalomyelitis, West Nile, human herpes simplex, Japanese encephalitis, and in immunocompromised humans or macaques, respectively, John Cunningham (JC) and Simian 40 (SV40) virus [10, 11, 21, 41, 47, 48]. Encephalitis is also a common neurological complication in patients with COVID-19, where we just begin to understand mechanisms of brain infection and post-infection sequelae [6, 18, 25, 28].

Comprehending the pathophysiological mechanisms of encephalitides is crucially important for developing more efficient treatment strategies, including neuroprotective interventions, particularly with regard to longterm damage. Although mortality markedly decreased and etiologies somewhat shifted, no clear long-term improvements in outcome were seen, as reported in a 40-year survey in Sweden [45]. Considering the prevailing neuropsychiatric disabilities following encephalitides, refining their outcome should be a highly prioritised research issue [45].

The present longitudinal study has been designed to analyze by innovative behavioral and magnetic resonance imaging tools in vivo, as well as by various ex vivo and *post mortem* tests, the downstream consequences of sudden pyramidal neuronal loss, including reactive neuroinflammatory and subsequent neurodegenerative processes. Experimentally eliminating in our model the numerous parallel cellular and molecular events characterizing infections, that make interpretation of causes and consequences often hard, will aid in better understanding mechanisms of neurodegeneration in general and thus in developing more targeted diagnostic instruments and therapeutic interventions.

## Materials and methods

### Mice

All animal experiments were approved by the local animal care and use committee (LAVES, Niedersächsisches Landesamt für Verbraucherschutz und Lebensmittelsicherheit, Oldenburg, Germany) in accordance with the German animal protection law. Mice were maintained in temperature- and humidity- controlled environment (~ 22 °C, ~ 50%), 12 h light/dark cycle (light on at 7am) with food and water ad libitum. Cages were enriched with wood-chip bedding and nesting material (Sizzle Nest, Datesand). All experiments were performed by investigators unaware of group assignment (‘fully blinded’). Behavioral testing-order was balanced between groups prior to experiments and randomized within groups via random selection by the blinded investigator.

C57BL/6 mice bearing the tamoxifen-inducible diphtheria toxin chain A allele were generated by crossing homozygous Neurod6^tm2.1(cre/ERT2)Kan^ (‘NexCreERT2’, [1]) with heterozygous Gt(ROSA)26Sor^tm1(DTA)Jpmb^ (‘Rosa26-eGFP-DTA’, [19]) resulting in double heterozygous inducible (‘DTA’) mice and heterozygous NexCreERT2 littermate (‘control’) mice lacking the DTA allele. Detailed PCR-based genotyping protocols are available upon request. Male transgenic mice were weaned at postnatal day 21 and separated by genotype to avoid inclusion effects or aggressive behavior against potentially affected animals. Experiments were performed on adult male mice (starting age 6–8 months) over a period of approximately 2 months.

### Tamoxifen induction

Tamoxifen (CAS#10540-29-1 T5648, Sigma-Aldrich) was dissolved in corn oil (C8267, Sigma-Aldrich) on injection days at 10 mg/mL. Dependent on the experimental cohort, mice received either 3 or 5 daily intraperitoneal injections of 100 mg tamoxifen/kg body weight.

### Blood sampling and high-mobility group box 1 (HMGB1) ELISA

Intermediate blood samples (100µL) were collected 2 weeks after the last tamoxifen injection from the retro-orbital sinus. Terminal blood (500µL) was sampled by cardiac puncture before transcardial perfusion. EDTA plasma aliquots were stored at −80 °C. Plasma HMGB1 concentrations were determined using a commercial HMGB1 ELISA assay (USC-SEA399MU-96, Biozol) according to the manufacturer’s instructions.

### Behavioral phenotyping

Experiments were performed during light phase in the following order: Open field, prepulse inhibition, Morris water maze, SocioBox and complex wheel running (the latter 2 only in 3 × tamoxifen mice).

#### Open field

To evaluate exploratory activity in a novel environment, mice were placed in the center of a gray circular Perspex arena (120 cm diameter, 25 cm height of outer wall). First, time to reach the outer wall (escape latency) was measured, followed by 7 min to freely explore the open field. When exceeding 180 s to reach the outer wall, mice were placed in the periphery zone to start 7 min exploration time. Behavior of the mice was recorded via tracking software (Viewer3, Biobserve). Analyzed parameters were covered distance and the time spent in the central, intermediate and peripheral zones of the open field. Animals were tested at the age of 27 (3 × tamoxifen) and 32 (5 × tamoxifen) weeks with light intensity of 120–140 lx in the center.

#### Prepulse inhibition (PPI) of the startle response

This paradigm has been described previously in detail [12]. In brief, to evaluate sensorimotor gating, animals were placed in small metal cages to prevent major movements. Cages were placed in sound attenuating cabinets (TSE Systems) on a sensor-attached platform to record movement. After habituation to 65 dB white noise, loudspeakers delivered acoustic stimuli to evoke startle reflexes. Stimuli of different intensity (70, 75, 80 and 120 dB) were used in a pseudo-randomized order. The amplitudes of the startle response were averaged within the various intensities for each mouse. PPI was calculated as percentage of the startle response using following formula: %PPI = 100–[(startle response after prepulse)/(startle amplitude after pulse only) × 100]. For analysis, data from non-performing mice (negative PPI) were excluded.

#### Morris water maze (MWM)

This test has been described previously in detail [12, 31]. Briefly, to measure spatial learning and memory, mice were placed in a circular tank (120 cm diameter, 60 cm height) filled with opaque water at room temperature (~ 22 °C) with the escape platform (10 cm diameter) approximately 1 cm submerged. Animals’ movement was recorded by video-tracking system (Viewer3, Biobserve). After 2 days of a visual platform task in which extra maze cues were covered by the tank walls, the visual cue (flag on platform) was removed, the platform was relocated and the water height was adjusted so that extra maze cues were visible. For 8 consecutive days, mice had to reach the “hidden” platform in 4 trials per day. Afterwards, the platform was removed and mice were observed during a single “probe trial”. Analyzed parameters were escape latency to platform and covered distance. Additionally, during probe trial, the time spent in, visits and latency to the target quadrant (formerly containing platform) and covered distance were measured. If groups showed no significant differences, the platform was once again relocated for another 4 days of reversal learning, followed by another probe trial. Except for the probe trials, each day consisted of 4 test runs with a maximum of 90 s each and an intertrial interval of 5 min. In between trials, mice were placed in single cages containing paper towels and standing on a heating pad to prevent hypothermia and overexertion of the mice. In absence of a platform to reach, both probe trials consisted of a single test run of 90 s. Animals were tested at the age of 28–30 (3 × tamoxifen) and 33–35 (5 × tamoxifen) weeks with light intensity of 120–140 lx.

#### SocioBox

A detailed description of this test has been published before [23]. Briefly, to evaluate social recognition and memory, 3 × tamoxifen mice were tested in the SocioBox at the age of 31–32 weeks with light intensity of 10–15 lx. They were placed in a circular apparatus (34 cm inner and 56 cm outer diameter) with 5 small boxes (“inserts”) within the outer wall. For each mouse, the SocioBox experiment consisted of 3 habituation sessions on 3 consecutive days and 2 exposures and 1 recognition trial on day 4. Within each trial, for the first 5 min, mice stayed in a circular partition (19 cm diameter) to prevent immediate exploring of the SocioBox (initiation stage). After lifting of this partition, mice were allowed to freely explore the SocioBox for another 5 min (interaction stage). While the 5 inserts in the outer ring were empty during all 3 habituation trials, they contained a mouse each for interaction purposes (“stimulus mice” or “stimuli”). For both exposure trials, the same 5 stimulus mice in the same position and order were used. For the final recognition test, one stimulus mouse was replaced by a new stimulus mouse (“stranger”), unknown to the test mouse. Perforated fronts of the inserts allowed limited interaction between test mouse and stimulus mice and front walls of each insert were exchanged after each trial. Age and sex matched C3H mice were used as stimuli, based on their reported robust social interaction in test situations [33]. Mouse movement was recorded via tracking software (Viewer3, Biobserve).

#### Thermography

This technique and data extraction/processing have been published previously in detail [39] and were slightly modified for the present application. In short, for SocioBox experiments (performed with 3 × tamoxifen mice), an A655sc infrared thermography camera (FLIR) was mounted 110 cm above the arena, recording images at 640 × 480 pixels and framerate of 2 Hz (habituation 3, exposure 1 + 2) and 5 Hz (memory test), via ResearchIR (FLIR Systems, Oregon, USA) and connected to a computer located in a separate room. Extraction of thermal data was done using OpenCV 4 in Python 3.6. Images were loaded and normalized to values between 0 and 255, with higher values meaning higher temperatures. The SocioBox arena in which the test mouse was allowed to move was defined as the relevant ROI for extracting thermal data. To keep only thermal data from the test mouse, a binary mask for whole body (including tail) was created by applying intensity thresholding and processing steps to decrease image noise. By doing so, one large cluster of connected pixels within this ROI could be detected, constituting the contour of the mouse. Due to the shape and temperature differences, the whole-body area could then be segmented into a central body and a distinct tail area, and the mean temperature of each of those 2 areas could be extracted. Analyzed parameters were temperature changes over time of test mice, latency and duration of interaction with stimulus mice and “strangers” and visits to interaction zones. The Centralization Index (ratio body/tail temperature = T ratio) was used as continuous temperature measure [39].

#### Complex wheel running

To stimulate neuronal activation-induced cFos expression in the hippocampus, mice were subjected to a complex running wheel (CRW) set-up for 4 h [43]. Mice were single housed in type III cages (42 × 26 × 18 cm, Tecniplast), equipped with CRW (TSE-Systems) characterized by randomized omitted bars [26, 27]. Mice were habituated to the experimental room and CRW for 2 h prior to dark phase. After dark phase onset, voluntary running was automatically tracked for 4 h via Phenomaster software (TSE-Systems) and the total running distance per mouse calculated. Mice were perfused with Ringer and 4% formaldehyde/PBS, 30 min after removal from the CRW set-up.

### Magnetic resonance imaging (MRI)

Mice (5 × tamoxifen) were anesthetized with ketamine and medetomidine (60 mg/kg and 0.4 mg/kg body weight), intubated and kept under 1.5% isoflurane by active ventilation with constant ventilation frequency of 85 breaths/min (Animal-Respirator-AdvancedTM, TSE-Systems). Inside the MR-System, mice were placed in a prone position with head fixed to a teeth and palate holder [7]. All MR measurements were performed at magnetic field strength of 9.4 T (Biospec®, Bruker BioSpin MRI, Ettlingen, Germany) comprising the following methods and acquisition parameters: High-resolution T2-weighted images (2D Rapid Acquisition with Relaxation Enhancement (RARE), TE/TR = 55/6000 ms, 8 echoes, spatial resolution 40 × 40 × 300 µm^3^), magnetization-transfer (MT) weighted images for volumetric analyses (3D fast low angle shot (FLASH), TE/TR = 3.4/15.2 ms, flip angle 5°, Gaussian-shaped off resonance pulse (off-resonance frequency 7.5 ppm, RF power 6µT), spatial resolution 100 µm isotropic), measurements of blood perfusion by dynamic susceptibility contrast (DSC) MRI (2D RF-spoiled radial multi-echo FLASH [32]: TE1,2,3 = 1/2.15/3.3 ms, TR = 9 ms, flip angle = 11°, spatial resolution = 150 × 150 × 900 µm^3^, 2 slices, 201 spokes, temporal resolution = 0.55 frames per second, 800 repetitions) and intra-voxel incoherent motion (IVIM) MRI (Stejskal-Tanner pulsed gradient spin-echo sequence, TE/TR = 19/2000 ms, 4 segments, δ = 2.5 ms, Δ = 10 ms, 19 b-values (10, 20, 30, 40, 50, 60, 70, 80, 110, 140, 170, 200, 300, 400, 500, 600, 700, 800, 900 s/mm^2^) applied in 3 orthogonal directions, spatial resolution 150 × 150 × 400 µm^3^).

### MRI data analyses

#### Volumetry

MT-weighted images were first converted to NIfTI and preprocessed through denoising and bias field correction [3] in order to create an unbiased anatomical population template using the python pipeline twolevel_ants_dbm (https://github.com/CoBrALab/). Nonlinear deformation fields of the extracted brains were then used to estimate voxel-wise Jacobian determinants. Student’s *t*-test was performed on the 3D Gaussian-smoothed maps of Jacobian determinants (FWHM 0.1 mm) for voxel-vise comparison of DTA and control mice. Q-values (false discovery rate (FDR) adjusted p-values) and the respective z-scores were calculated using the 3dFDR function of AFNI [9]. To visualize significant volume reductions in DTA mice z-scores smaller − 2.57 corresponding to a FDR of less than 1% were overlaid on the study template. In order to quantify the volume of selected brain regions, regions of interest (ROIs) including third and lateral ventricles, cerebrum (without hippocampus, ventricles and olfactory bulb), hippocampus, and cerebellum were determined on the study template by manual segmentation using the software package AMIRA (Visage Imaging GmbH, Berlin, Germany). ROIs were then retransformed into the subject space, individually inspected, and, if required, manually corrected. Finally, the respective volume information was extracted.

#### IVIM

Diffusion weighted images were co-registered (translation only, imregister function, Matlab 2018b, Natock, MA) and the geometrical mean of the diffusion directions, the apparent diffusion coefficient (ADC) was calculated considering only those images obtained with b-values greater than 200 s/mm^2^. The ADC was then used to estimate the perfusion fraction (f) and the pseudo-diffusion coefficient (D*) by utilizing all acquired b-value-images [13].

#### DSC-MRI

Maps of R2*-relaxation rate (1/T2*) were estimated assuming a mono-exponential TE-dependent signal decay. A gamma variate function was fitted to the R2*-time curve of the contrast agent bolus-injection in order to estimate the cerebral blood volume (CBV) and the mean transit time (MTT) from which the cerebral blood flow (CBF) was calculated (CBF = CBV/MTT).

### Measurements assessing blood–brain-barrier integrity

Blood–brain barrier (BBB) integrity along with brain water content and dry brain mass was evaluated as previously described [5, 34]. Briefly, mice received intravenous injections of Evans blue (50 µg/g body weight, E2129, Sigma-Aldrich) and sodium fluorescein (200 µg/g body weight, F6377, Sigma-Aldrich). After 4 h, mice were anesthetized and transcardially perfused. Brains were collected, frozen on dry ice, weighted and lyophilized. After lyophilization, dehydrated brains were weighted to obtain dry brain mass and to calculate brain water content. Afterwards, tracers were extracted from hemispheres with formamide and quantified in triplicates on a fluorescent microscope (Observer Z2, Zeiss). The concentrations of tracers were calculated using a standard curve and normalized to controls.

### Blood flow cytometry

EDTA-blood was collected from the orbital sinus 2 weeks after last tamoxifen injection. Per mouse, 50µL blood were diluted in 50µL PBS and overlaid on 100µL lymphocyte separation medium (1077, PromoCell). After centrifugation, peripheral blood mononuclear cells were isolated, washed and stained for 15 min at 4 °C with the following antibodies: PECy5 anti-CD4 (1:1000, clone H129.19, Biolegend), PECy7 anti-CD8 (1:500, clone 53–6.7, Biolegend), BV510 anti-B220 (1:500, clone RA3-6B2, Biolegend) and PerCpCy5.5 anti-CD11b (1:100, clone M1/70, Biolegend). After staining, cells were washed, suspended in 200µL PBS containing 2% bovine serum albumin (#8076.3, Roth), and filtered through 40 µm cell strainers. Samples were measured on a FACSAria Sorp (BD). Number of lymphocytes were determined using forward and side scatter. Frequency of T-helper cells (CD4^+^,CD8^−^), cytotoxic T-cells (CD8^+^, CD4^−^), B cells (B220^+^, CD11b^−^, CD8^−^, CD4^−^), and myeloid cells (CD11b^+^, B220^−^, CD8^−^, CD4^−^) were determined as percentage of total lymphocytes.

### Brain flow cytometry

Mice (3 × tamoxifen) were anesthetized with Avertin (1.36% 2,2,2,-tribromoethanol in ddH_2_O; 24µL/g body weight) and transcardially perfused with 40 mL Ringer solution (B.Braun). Brains were stored on ice in 10% fetal bovine serum (FBS, #10500–064 Thermo)/DMEM (#41965 Thermo) until all brains were collected. Olfactory bulbs and brain stems were removed and brains mashed through 70 µm cell strainers. To remove myelin, cells were suspended in isotonic Percoll (17-0891-01, GE Healthcare) to a final concentration of 30% and centrifuged. Cells were washed with FACS buffer (2% FBS, 10 mM EDTA in PBS) and filtered through 40 µm cell strainers. Fc-receptors were blocked for 10 min at 4 °C with anti-mouse CD16/32 antibodies (1:100, 14–0161, eBioscience). Cells were stained for 30 min at 4 °C with the following antibody mix: APC anti-CD45 (1:200, clone 104, BioLegend), PE anti-CD11b (1:200, clone M1/70, BioLegend), PECy5 anti-CD4 (1:1000, clone H129.19, BioLegend), PECy7 anti-CD8 (1:500, clone 53–6.7, Biolegend), APCCy7 anti-CD19 (1:200, clone 6D5, BioLegend), PerCP-Cy5.5 anti-CD138 (1:200, clone 281–2, BioLegend). After staining, cells were washed and suspended in 400µL FACS buffer. Per sample, 100µL APC quantification beads (#340487, BD) were added. Samples were measured on a FACSAria Sorp (BD). Cell numbers were corrected for the number of recorded APC beads. Leukocytes (CD45^high^, CD11b^−^), microglia (CD45^low^, CD11b^high^) and macrophages (CD45^high^, CD11b^high^) were quantified within single cell gate determined by forward and side scatter. CD4^+^ T-cells and CD8^+^ T-cells were quantified within leukocyte gate. CD19^+^ B-cells and CD138^+^ plasma cells were quantified in CD4^−^ CD8^−^ leukocyte gate.

### Histology

Mice were anesthetized with Avertin, transcardially perfused with Ringer (B.Braun) and subsequently 4% formaldehyde/PBS. Brains were collected, post-fixed in 4% formaldehyde/PBS for 12 h, dehydrated in 30% sucrose/PBS for 48 h, embedded in optimal cutting medium (Tissue-Tek, #4583, Sakura) and frozen on dry ice. Frozen brains were cut into 30 µm coronal sections on a cryostat (CM1950, Leica) and stored at −20 °C in anti-freeze medium (25% ethylene glycol/25% glycerol/PBS). Quantifications were performed using 4–6 hippocampi from 2–3 sections per mouse. Sections were selected in regularly spaced intervals (every 300 µm) between Bregma coordinates −1.34 to −2.24 mm. Free-floating frozen sections were blocked and permeabilized for 1 h at RT with 5% normal horse serum (NHS, 26,050–088, Thermo) in 0.5% Triton X-100/PBS, incubated overnight at 4 °C with primary antibodies and subsequently stained with corresponding fluorescently-labeled secondary antibodies for 2 h at RT. Nuclei were stained for 10 min at RT with 0.2 µg/mL 4′,6-diamidino-2-phenylindole in PBS (DAPI, D9542, Sigma-Aldrich) and sections were mounted on SuperFrost®-Plus slides (J1800AMNZ, Thermo) with Aqua-Poly/Mount (#18606, Polysciences). The following primary antibodies were used: Mouse anti-GFAP (1:500, NCL-GFAP-GA5, NovoCastra), rabbit anti-Iba1 (1:1000, #019–19741, Wako), rabbit anti-cFos (1:1000, #226003, Synaptic Systems), guinea pig anti-parvalbumin (1:1000, #195004, Synaptic Systems). Corresponding secondary antibodies included: Alexa Fluor 555 anti-rabbit (1:1000, A21428, Thermo), Alexa Fluor 647 anti-mouse (1:1000, A31571, Thermo) Alexa Fluor 633 anti-guinea pig (1:1000, A21105, Thermo). For Fluorojade C staining (AG325, Sigma) of dying neurons, sections were incubated in 0.06% potassium permanganate solution for 10 min. Following a 1 min water rinse, tissue was transferred for 10 min to a 0.0001% solution of Fluorojade C, dissolved in 0.1% acetic acid. Slides were rinsed with ddH2O and dried at 60 °C. Overview images of whole brain sections were obtained on Eclipse-TI 2 epifluorescence microscope (Nikon), equipped with 4 × objective (4x/0.2 NA PLAN APO #MRD00045, Nikon). For quantification of Iba1^+^ cells and GFAP^+^ area fraction by densitometry, 1 µm thick optical sections of hippocampi were acquired as tile scans on a confocal laser scanning microscope (LSM 880, Zeiss), furnished with a 40 × oil objective (40x/1.4 NA Plan-APOCHROMAT, #420762–9900, Zeiss). For quantification of parvalbumin^+^ and cFos^+^ neurons, 2 µm thick optical sections of hippocampi were acquired as tile scans using the same microscope, equipped with a 20 × air objective (20x/0.8 Plan-APOCHROMAT, #420640–9903 Zeiss). Image acquisition parameters were kept constant within experiments. Quantifications and image processing were performed with FIJI-ImageJ software (Schindelin et al., 2012). Iba1^+^ cells (mostly microglia), parvalbumin^+^ cells (inhibitory neurons) and cFos^+^ neurons were manually counted. GFAP^+^ area fraction (GFAP^+^ area/total area of region of interest) was quantified densitometrically upon uniform thresholding and fold change to control animals was calculated. Atrophy in regions of interest (CA1, CA3, dentate gyrus) was determined through manual segmentation. Resulting areas were normalized to the respective average of control animals. CA2/CA3 region is referred to as CA3 in text and figures. Cell counts were normalized to quantified areas. Data obtained from 4 to 6 hippocampi/mouse was averaged for analysis.

### Statistical analysis

Statistical analyses were performed using Prism9 software (GraphPad Software). Results are presented as mean ± standard deviations (SD), unless otherwise stated. Normal distribution of data was assessed using the Shapiro–Wilk test with an alpha error of 0.05. Dependent on data distribution, 2-tailed unpaired Welch’s corrected *t*-tests or Mann–Whitney U-tests were used to compare groups. Repeated measure data was analyzed using mixed-model ANOVA. P values < 0.05 were considered statistically significant.

## Results and discussion

### Sudden pyramidal neuronal loss by induced *diphtheria* toxin expression for sterile modeling of viral-like gray matter encephalitis

**Fig. 1:**
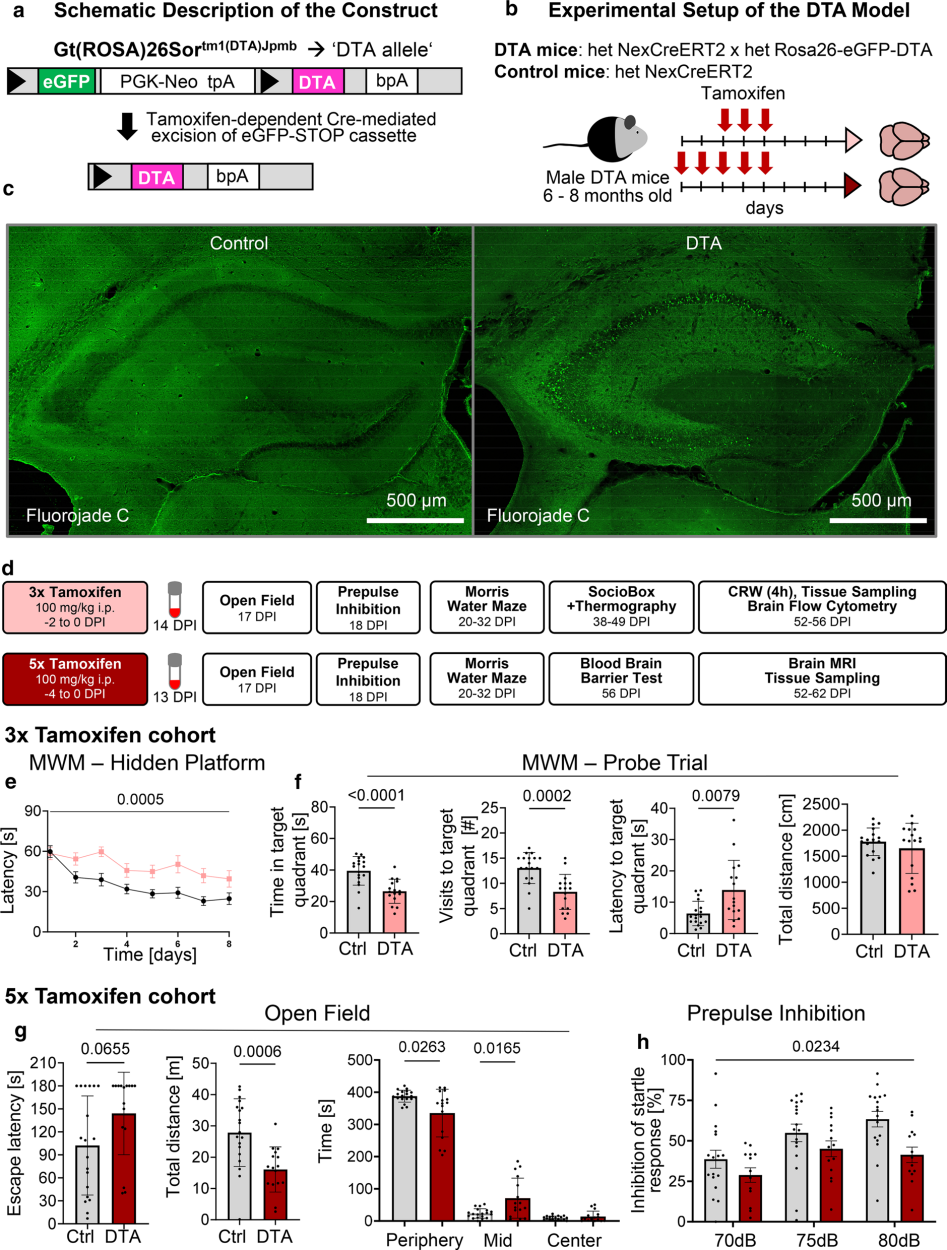
Effects of sterile gray matter encephalitis, induced by pyramidal neuronal ablation, on diverse behavioral paradigms. **a** Schematic description of the diphtheria toxin chain A (DTA) allele. Tamoxifen-dependent Cre-mediated excision of a STOP-cassette leads to expression of DTA, subsequent inhibition of protein synthesis, and cell death. **b** Genotype of the DTA model targeting pyramidal neurons and tamoxifen dosing scheme. Cohorts induced with 3 × tamoxifen included 16 DTA and 18 control mice. The 5 × tamoxifen cohort comprised 16 DTA and 19 control animals. **c** Fluorojade staining of hippocampal sections at 1 week after 5 × tamoxifen induction. Note the massive acute neurodegeneration and pyramidal neuronal loss. **d** Experimental outline illustrates groups, experiments and timeline of testing (DPI = days post induction, CRW = complex running wheel, MRI = magnetic resonance imaging). **e** Cognitive testing in Morris water maze (MWM), with hidden platform task showing significantly inferior learning curve (latency to reach the platform) of 3 × DTA mice (light red) compared to control (black); repeated measures mixed-model ANOVA; mean ± SEM. **f** Spatial memory testing in the probe trial indicating significantly less time spent in target quadrant (TQ, formerly containing hidden platform), less visits to TQ and longer latency to reach TQ of 3 × DTA mice. Total swimming distance of both groups did not differ. Data presented as mean ± SD. **g** Testing 5 × tamoxifen groups for anxiety and exploratory behavior in the open field showed a tendency of increased escape latency from center towards periphery. DTA compared to control mice covered less distance, spent less time in the periphery and more time in “mid” (intermediate zone between center and periphery). Time in center showed no differences between both groups (*p* = 0.9546). Data presented as mean ± SD. **h** 5 × DTA compared to control mice displayed a decreased prepulse inhibition; repeated measures mixed-model ANOVA; mean ± SEM

To comprehensively study distinct features of moderate brain inflammation that affects primarily gray matter, we targeted pyramidal neurons. By inducible *diphtheria* toxin expression in these cells, we generated a sterile, spatially and temporally defined, moderate experimental encephalitis in 6–8 months old male NexCreERT2xRosa26-eGFP-DTA (= ‘DTA’) mice [1, 8, 19]. Tamoxifen dosing in this mouse line controls the amount of cell death. After a series of dose-titrating pilot experiments, we here selected a 3-day (3x) versus 5-day (5x) tamoxifen injection design (Fig. 1a, b). Acute hippocampus overview images, taken from trial mice at 1 week after 5 × tamoxifen induction, demonstrate by fluorojade staining the acute neurodegeneration/pyramidal neuronal loss (Fig. 1c). Using the 3 × versus 5 × tamoxifen design, we performed a longitudinal ~ 60 day analysis of these mice, which included behavioral experiments to target hippocampal and (pre)frontal cortex functions, with both established (MWM, PPI) and novel (SocioBox, thermography) paradigms, several sophisticated MRI readouts, BBB testing, flow cytometry analyses of brain and blood, plasma HMGB1 measurements as circulating inflammation marker, as well as histological quantifications, including a neuronal functionality readout (immediate early gene cFos induction after brief complex wheel running). In order to reduce the necessary number of animals (RRR principle), not all experiments were performed in mice of both induction schedules (Fig. 1d).

### Behavioral testing reveals manifold disturbance of brain functions in DTA mice

After destruction of substantial numbers of pyramidal neurons, hippocampal learning and memory, determined by the classical MWM, was highly significantly affected in encephalitis mice of both induction schemes (Fig. 1e-f and Tables 1,2). In the 5 ×, but not the 3 × tamoxifen group, the open field performance reflected behavioral pathology with reduced overall mobility and dampened natural drive to prefer the periphery (Fig. 1g; Tables 1,2). Both escape latency and enhanced time spent in the middle zone may additionally be related to the slower motion/reduced total distance. The PPI data in 5 × mice demonstrated also (pre)frontal network dysfunction (Fig. 1h; Table 2).

**Table 1:**
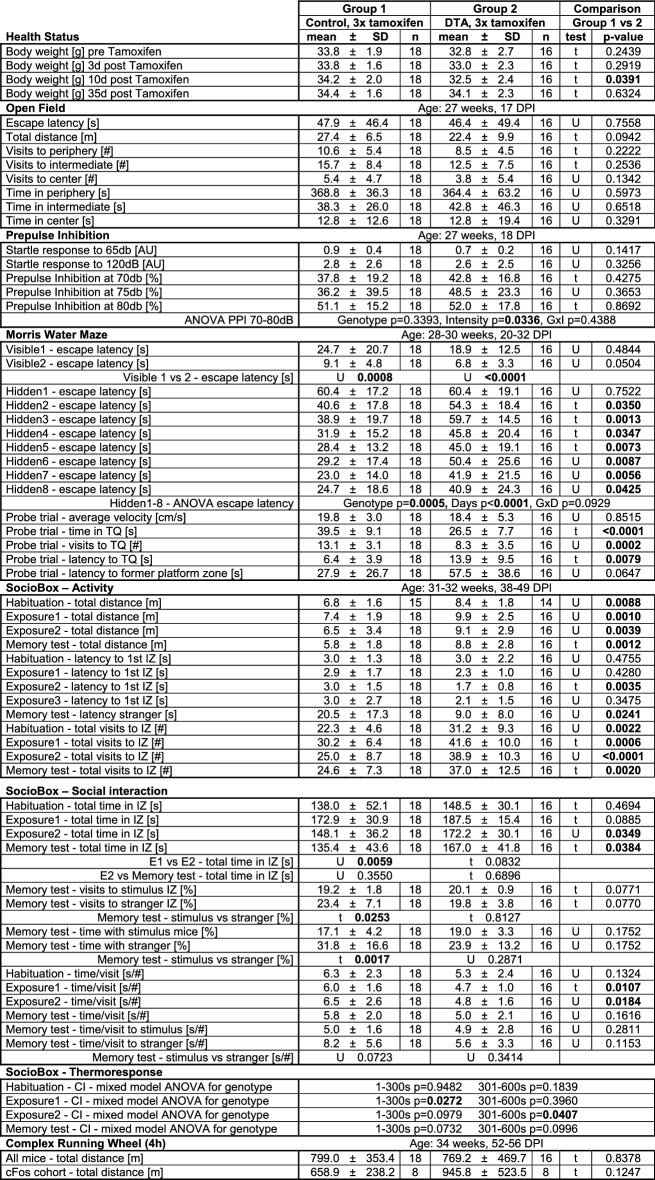
**Results of behavioral testing of the 3 × tamoxifen induced encephalitis mice** (t = 2-sided Welch's corrected *t*-test; U = 2-sided Mann–Whitney U-test. *TQ* target quadrant, *IZ* interaction zone, *CI* centralization index)

**Table 2:**
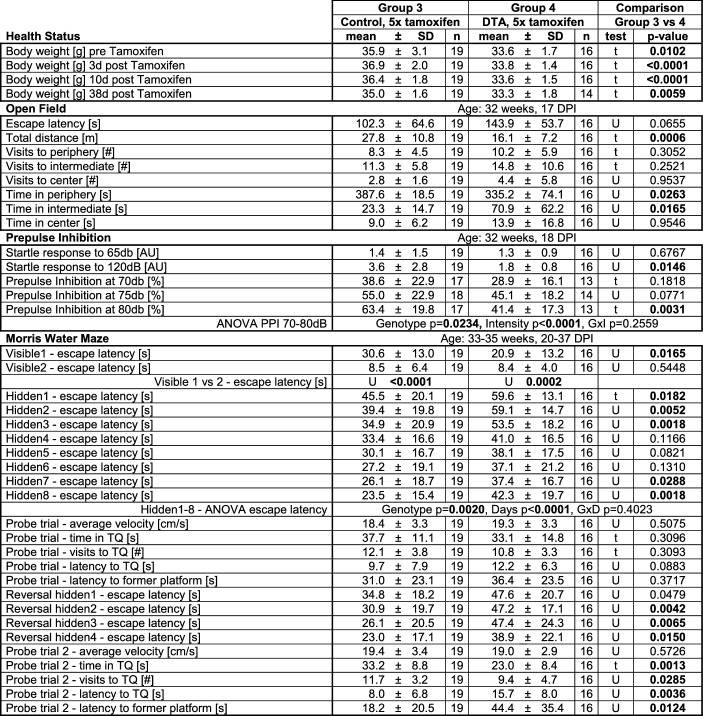
**Results of behavioral testing of the 5 × tamoxifen induced encephalitis mice** (t = 2-sided Welch's corrected *t*-test; U = 2-sided Mann–Whitney U-test)

**Fig. 2:**
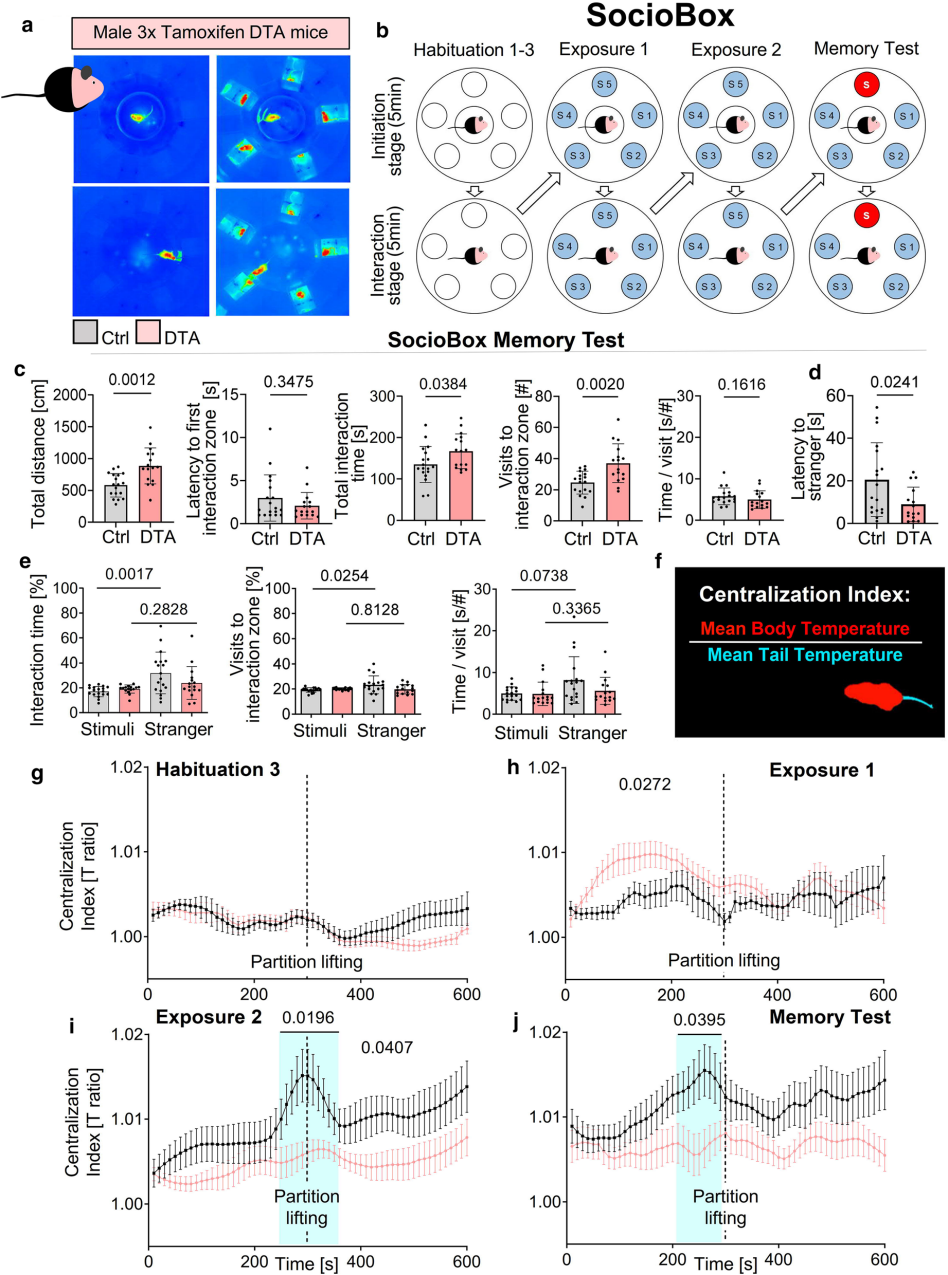
Consequences of defined gray matter encephalitis on social recognition memory and thermoregulation in the SocioBox. **a** SocioBox experiments were performed using the 3 × tamoxifen cohort (DTA n = 16, control n = 18). Infrared thermography recordings show test mouse during habituation 3 in an otherwise empty SocioBox (left side) and during exposure to 5 stimulus mice (right side). Note the presence of the divider (partition) in the upper row that is lifted in the lower row. **b** Schematic outline of the 6 SocioBox trials. Habituations 1–3 were performed on 3 consecutive days, while exposure 1–2 and memory test were all performed on day 4. Each trial consisted of an initiation and interaction stage (5 min each; separated by divider lifting). During exposure, each test mouse (middle) was confronted with 5 stimulus mice (stimuli; S1-S5, light blue). For the memory test, 1 stimulus mouse was exchanged with a new mouse (“stranger”, S, red) unknown to the test mouse. **c** General activity during memory test was increased in DTA compared to control mice (total distance, total interaction time, visits to interaction zone), whereas the latency to first interaction zone and time/visit were similar. **d** DTA mice were significantly faster to approach the stranger than control mice. **e** Evaluation of social recognition memory in the SocioBox memory test: Comparing the interaction of DTA and control mice with their conspecifics showed that control mice spent more time with the stranger than with the stimulus mice and undertook more visits to the stranger than to stimulus mice. DTA mice displayed no differences in these parameters, indicating a lack of distinction between stimulus mice and stranger; for clarity, parameters given in % total interaction time and average of time spent with all 4 stimulus mice shown. Time per visit to stranger over stimuli tended to be increased in controls but not DTA mice. **f** Centralization Index (CI) was calculated by mean body temperature divided by mean tail temperature for every frame of recording. **g–j** CI changes of the test mice during SocioBox trials. Dotted line indicates partition lifting after 300 s; repeated measure mixed-model ANOVA, mean ± SEM presented. **g** During habituation 3, there were no CI differences between both groups, neither before (*p* = 9482) nor after partition lifting (*p* = 0.1839). **h** During first half of exposure 1, DTA mice showed a higher CI than controls, but no difference after partition lifting (*p* = 0.3960). **i** First half of exposure 2 showed no significant differences between both groups (*p* = 0.0979), but revealed higher CI of control mice during second half (*p* = 0.0407). Interestingly, control mice presented increased CI values towards partition lifting (250-350 s, light blue field). **j** During the memory test, differences between groups in first (*p* = 0.0732) or second half (*p* = 0.0996) were not significant, but controls exhibited higher CI values in anticipation of partition lifting (210-290 s, *p* = 0.0395, light blue field)

On top of these expected behavioral changes, we addressed novel readouts of social behavior, suspected to reflect CA2/CA3 destruction in our model, and known to play a role in human viral encephalitis [4, 35]. We tested in the 3 × tamoxifen mice social recognition memory, employing our recently developed SocioBox, and combined it with thermography (Fig. 2a-b; Table 1). The SocioBox test is superior to other presently available social recognition tests, successfully addressing multiple social contacts in parallel [23, 39]. We noted overall hyperactivity in the SocioBox memory test displayed by DTA mice, from total distance traveled to social interactions, including increased total interaction time and visits to the interaction zone, combined with a trend of shorter initial latency to interaction zone and time/visit (Fig. 2c). Surprisingly at first view, the latency to meet the stranger was reduced in DTA mice (Fig. 2d). The following readouts, however, clarified that this reduced latency was by no means a signal of better social memory. Comparing the behavior towards acquainted conspecifics (stimuli) versus the stranger confirmed that DTA mice were not able to distinguish between them, whereas control mice discriminated well (Fig. 2e), explicitly indicating social cognition deficits of encephalitis mice. For better comprehension of this novel test paradigm, we provide supplementary videos, showing a healthy control mouse. The video on the left illustrates the centralization index. The video on the right demonstrates the SocioBox with the test mouse in the middle, surrounded by 5 stimulus mice. The stranger mouse is encircled. Note the experimentator’s arm in the middle of the video when partition lifting takes place.

### Thermography as convenient measure of autonomic dysfunction in DTA mice

We had reported previously that the SocioBox test with its forced and inescapable social interaction delivers substantial stress to healthy mice, even leading to the development of social avoidance, and thus constitutes a terminal test in behavioral batteries [39]. The distinct thermographic changes in this situation, resulting from stress-related blood flow alterations via reactive vasoconstriction and vasodilation, respectively, suggested the concomitant screening of body/tail temperature during SocioBox performance in our model. Interestingly, upon CNS inflammation, both on experimental autoimmune encephalitis in rats and human limbic encephalitis, evidence of thermoregulatory and autonomic dysfunction has been reported [2, 46].

In our thermography approach, the Centralization Index (ratio body/tail temperature = T ratio; see supplementary video) serves as whole body stress readout and convenient measure of potential autonomic dysfunction. Indeed, encephalitis mice (3 × tamoxifen) showed altered thermo-responses as compared to controls. Whereas during habituation 3, no appreciable dissociation in the thermo-curves appeared yet, DTA mice revealed higher centralization values in exposure 1 before partition lifting, perhaps pointing to an initially higher ‘non-specific’ agitation/stress. In both exposure 2 and memory test, the DTA mice thermo-responded in an overall blunted fashion compared to controls. In fact, a highly interesting thermo-pattern was observed in healthy mice that showed from exposure 1 over 2 to memory test a strong thermo-response, likely reflecting rising anticipation of partition lifting (Fig. 2f–j). The complete lack of this amazingly robust and fast experience-induced thermo-response in the sense of an ‘autonomically expressed short-term memory’, uncovered altered vasoactivity in peripheral and core body regions as indication of autonomic dysfunction and/or compromised short-term memory in DTA mice.

### Brain dimensions and structural changes of DTA mice in high-resolution MRI

**Fig. 3:**
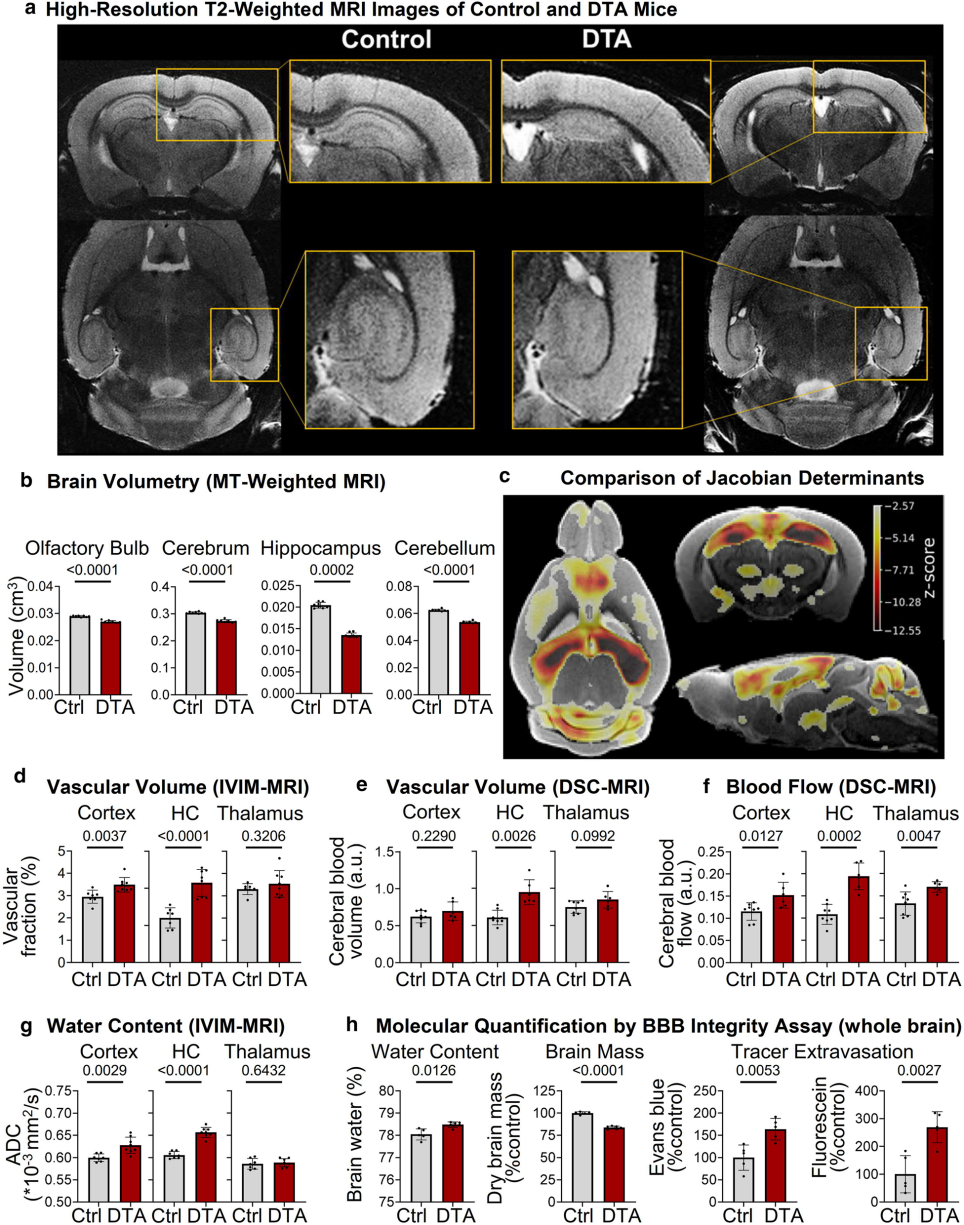
Magnetic resonance imaging 2 months after 5 × tamoxifen induction. **a** High-resolution T2-weighted MRI images of control and DTA mice. DTA mice displayed a reduction of the hippocampal dimensions in high-resolution T2-weighted MRI in vivo (top coronal view, bottom axial view). Dentate gyrus and CA region exhibited a clear rarefaction, making these regions in DTA mice almost undetectable. **b** Volumetric comparison of various brain regions. DTA mice showed atrophy of olfactory bulb, whole cerebrum (without hippocampus and ventricles), hippocampus, and cerebellum. **c** Comparison of Jacobian determinants. Maps of z-score derived from the comparison of Jacobian determinants, overlaid on the study template, revealed lower volume of the cortex (particularly in the region of layer V), the hippocampus and its projection regions in DTA mice compared to controls. A z-score lower than -2.57 corresponds to a false discovery rate lower than 1%. **d–f** Comparison of vasculature and perfusion by IVIM-MRI and DSC-MRI. DTA mice showed higher cerebral blood volume in the hippocampus as shown independently by IVIM-MRI **d** and DSC-MRI **e**. In addition, the vascular volume fraction was enhanced in the cortex of DTA mice. The cerebral blood flow **f** was increased in all analyzed brain regions. **g** Comparison of brain water content, determined by IVIM-MRI: Apparent diffusion coefficient (ADC) was increased in cortex and hippocampus of DTA mice, indicating higher water content in brain regions particularly affected by diphtheria toxin expression; MRI data from 6–8 mice/group, mean ± SD presented. **h** Molecular quantification of brain water, dry brain mass and blood brain barrier function in additional DTA and control mice at 2 months after encephalitis induction. Using this independent method, brains of DTA mice showed again higher water content and decreased brain mass. Increased extravasation of Evans blue and fluorescein indicate lasting blood–brain-barrier dysfunction; data from 5 mice/group, mean ± SD given; 2-tailed unpaired Welch’s corrected *t*-tests or Mann–Whitney U-tests. All experiments in Fig. 3 were performed with 5 × tamoxifen DTA mice

DTA mice (5 × tamoxifen) displayed a significant shrinkage of the hippocampal area shown by in vivo high-resolution T2-weighted MRI. Dentate gyrus and CA region exhibited a clear rarefaction, rendering these regions almost undetectable (Fig. 3a, top axial view, bottom transversal view). Volumetrical analysis of MT-weighted MRI demonstrated highly significant reductions in essentially all brain areas, including olfactory bulb, cerebrum, hippocampus and cerebellum (Fig. 3b). Maps of z-score derived from the comparison of Jacobian determinants and overlaid on the study template revealed significantly lower volume of the cortex (in particular in the region of layer V), the hippocampus and its projection areas in DTA mice compared to controls. A z-score lower than −2.57 corresponds to a false discovery rate of lower than 1% (Fig. 3c).

DTA mice had a higher vascular volume in the hippocampus as shown independently by both IVIM-MRI (vascular volume fraction) and DSC-MRI (cerebral blood volume). In addition, the vascular volume fraction was increased in the cortex of DTA mice. This phenomenon is highly interesting and will require deeper mechanistic search via experimental approaches in the future, as any clear insight from the literature [24] or from the present data is still lacking. Importantly, cerebral blood flow (DSC-MRI) was increased in all analyzed brain regions (Fig. 3d–f). Taking these findings together, we suggest a working model where the enhanced vascular volume, in parallel to neural tissue loss in this sterile encephalitis, reflects a ‘relative’ increase in vascular density rather than simple vessel dilatation. This would also reconcile with the observed increase in cerebral blood flow.

The apparent diffusion coefficient (ADC) was augmented in cortex and hippocampus but not thalamus of DTA mice, indicating higher water content in brain regions directly affected by the toxin (Fig. 3g). This finding was independently verified in the BBB integrity assay [5, 34], showing increased water content and reduced brain mass. At the same time, this assay yielded distinct tracer extravasation, both of Evans blue and fluorescein, proving BBB leakage (Fig. 3h).

### Lasting neuroinflammation following pyramidal neuronal death in DTA mice

**Fig. 4:**
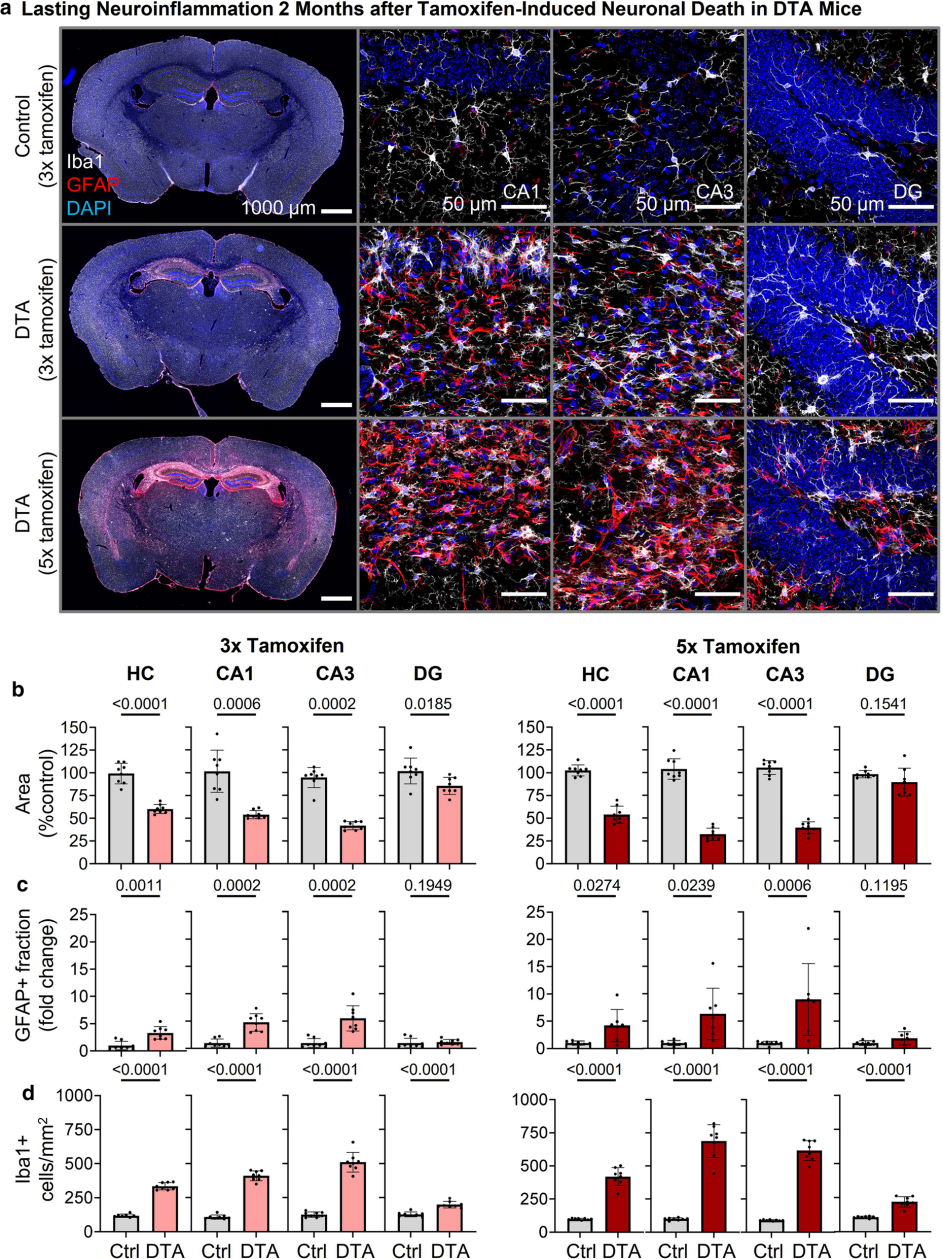
Histological analysis of neuroinflammatory readouts in the hippocampus 2 months after 3 × versus 5 × tamoxifen induction. **a** Representative coronal sections demonstrating persistent neuroinflammatory changes in DTA mice dependent on the tamoxifen dose. In the hippocampus of DTA mice, changes include increased microglia (Iba1^+^ cells, white) and GFAP (red) density as well as apparent changes in morphology. High-resolution images of CA1, CA3 and dentate gyrus (DG) regions were acquired as 10 µm Z-stacks and are displayed as maximum-intensity projections. **b** To assess atrophy in hippocampal regions, 4–6 hippocampi per mouse (within Bregma -1.34 mm and -1.94 mm) were manually segmented and areas of respective regions normalized to the mean of control mice. DTA mice after both tamoxifen doses showed strong atrophy in whole hippocampus (HC) and particularly its CA regions, whereas the DG was only weakly affected. **c** Evaluation of astrogliosis in DTA compared to control mice. Prominent astrogliosis was observed in HC and CA regions of DTA mice in both tamoxifen dose groups, while the DG remained relatively unaffected. The GFAP^+^ area fraction was determined densitometrically upon uniform thresholding, and fold changes were calculated based on the mean of control mice. **d** Quantification of Iba1^+^ cells (microglia). DTA compared to control mice showed increased microglia numbers in all hippocampal regions. Data from 7–8 mice/group displayed as mean ± SD; 2-tailed unpaired Welch’s corrected *t*-tests or Mann–Whitney U-tests

Histological analysis demonstrated lasting prominent micro- and astrogliosis in hippocampus and cortex of DTA mice, more pronounced after 5 × compared to 3 × tamoxifen (Fig. 4a). Quantifications validated again a clear reduction in the areas of whole hippocampus as well as of CA1 and CA3 subfields. The dentate gyrus was less noticeably affected (Fig. 4b). Similarly, the highest density of GFAP^+^ area and strongest increases in Iba1^+^ cell numbers were seen in total hippocampus, CA1 and CA3, but less in dentate gyrus (Fig. 4 c-d).

The prolonged neuroinflammatory response upon DTA-induced neuronal death is consistent with a mouse model, in which DTA expression was coupled to regulatory elements of Camk2α in a tetracyclin-responsive fashion [49], although kinetics of DTA induction, severity and regional selectivity expectedly differ between the 2 models. Interestingly, in this earlier mouse model, some of the neuroinflammatory responses, including reactive microglia, have been dampened upon microglia depletion and repopulation via CSF1R inhibitor treatment and withdrawal [38], demonstrating the importance of such animal models for developing translationally relevant treatment strategies.

To evaluate neuronal activation and interneuron density after selective ablation of pyramidal neurons in DTA mice, cFos expression and parvalbumin^+^ interneuron numbers were assessed. In fact, the toxin is tamoxifen-inducibly expressed by glutamatergic pyramidal neurons, which are preferentially destroyed. In contrast to the disease *Diphtheria*, however, only the catalytically active fragment A is expressed in our genetic model, while fragment B was purposely omitted to prevent receptor-mediated entry to neighboring cells [1, 8, 19]. Hence, cFos activity in inhibitory neurons should not have been directly affected by the toxin.

**Fig. 5:**
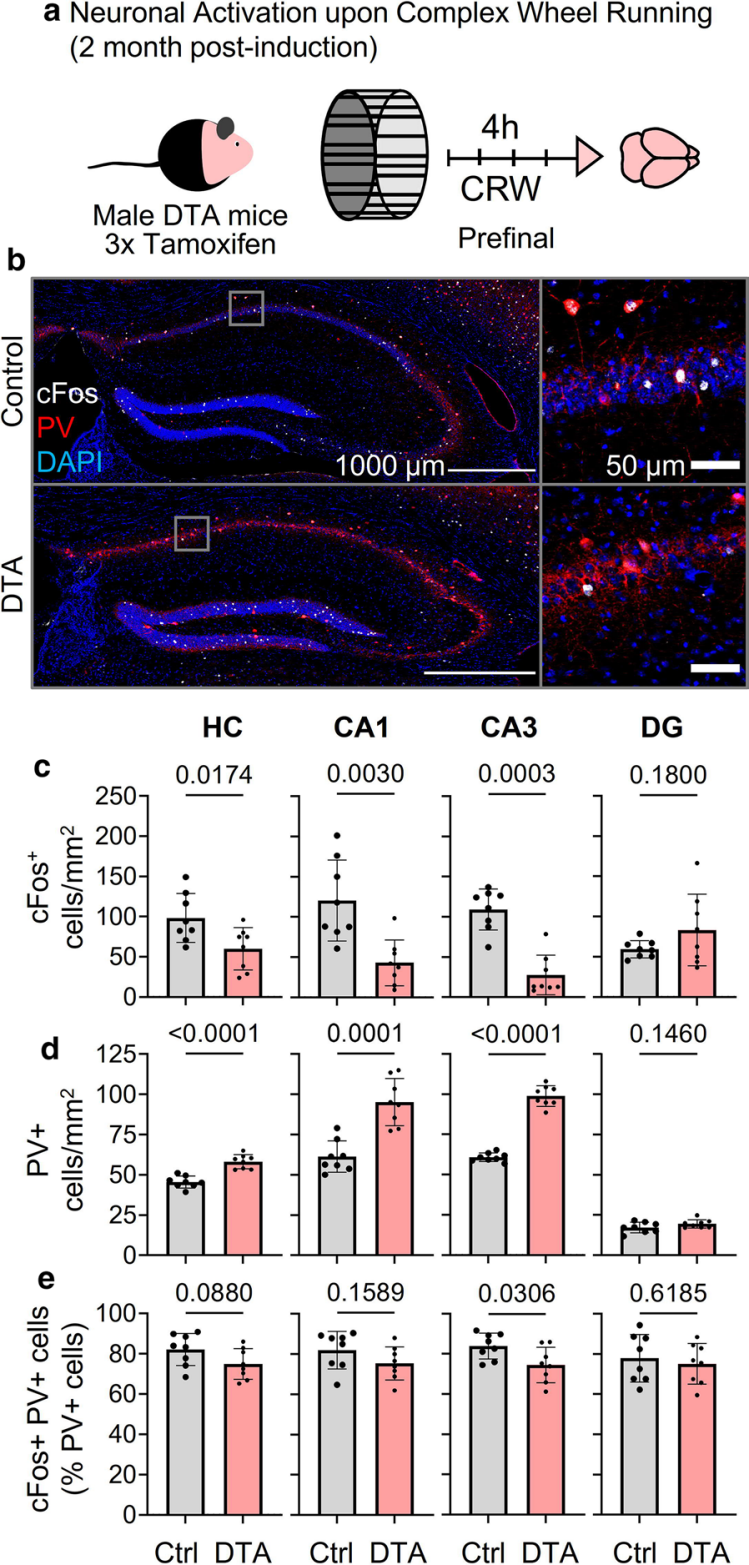
Indicators of neuronal activity and circuitry in the hippocampus upon complex wheel running. **a** Schematic experimental outline. Approximately 2 months after 3 × tamoxifen induction mice were challenged for 4 h exposure to voluntary complex wheel running to induce neuronal activation. **b** Representative images of cFos (white) and PV (red) double staining in control and DTA mice. **c** Quantification of total number of cFos^+^ cells in hippocampal regions upon CRW. DTA mice showed significantly reduced numbers of cFos^+^ cells in HC and its CA regions, whereas the dentate gyrus (DG) remained unaffected. **d** PV^+^ interneuron numbers 2 months after ablation of pyramidal neurons. DTA mice displayed increased PV^+^ interneuron numbers in HC and its CA regions, but not in DG. **e** Evaluation of PV^+^ interneuron activation upon CRW. PV^+^ interneurons were less frequently activated (evaluated by cFos expression) in CA3 region of DTA mice, with similar tendency in CA1. DG was again similar between both groups. Data from 8 mice/group displayed as mean ± SD; 2-tailed unpaired Welch’s corrected *t*-tests or Mann–Whitney U-tests

Indeed, determination of cFos expression after brief complex wheel running as neuronal activity readout confirmed the expected decrease of immediate early gene expression in whole hippocampus, CA1 and CA3 of DTA mice, due to the ablation of pyramidal neurons. In contrast, cFos^+^ cells in dentate gyrus did not differ from controls, again underlining the widely intact situation of this region (Fig. 5a–c). We note that both DTA and control mice performed comparably in this short-term CRW exposure (running distance: 659 ± 238 m versus 946 ± 523 m, respectively, *p* = 0.1893). The number of parvalbumin^+^ interneurons normalized to the respective hippocampal area showed highly significant ‘relative’ increases, consistent with circuit adjustments in the sense of altered inhibition. The cFos^+^ interneurons among them, however, tended to be decreased in whole hippocampus and CA subfields, pointing perhaps to mildly compromised cellular activatability. Again, the dentate gyrus was spared in DTA mice and appeared comparable to controls (Fig. 5d, e).

### Flow cytometry shows clear alterations in the brain immune compartment following pyramidal neuronal death in absence of measurable effects in blood

**Fig. 6:**
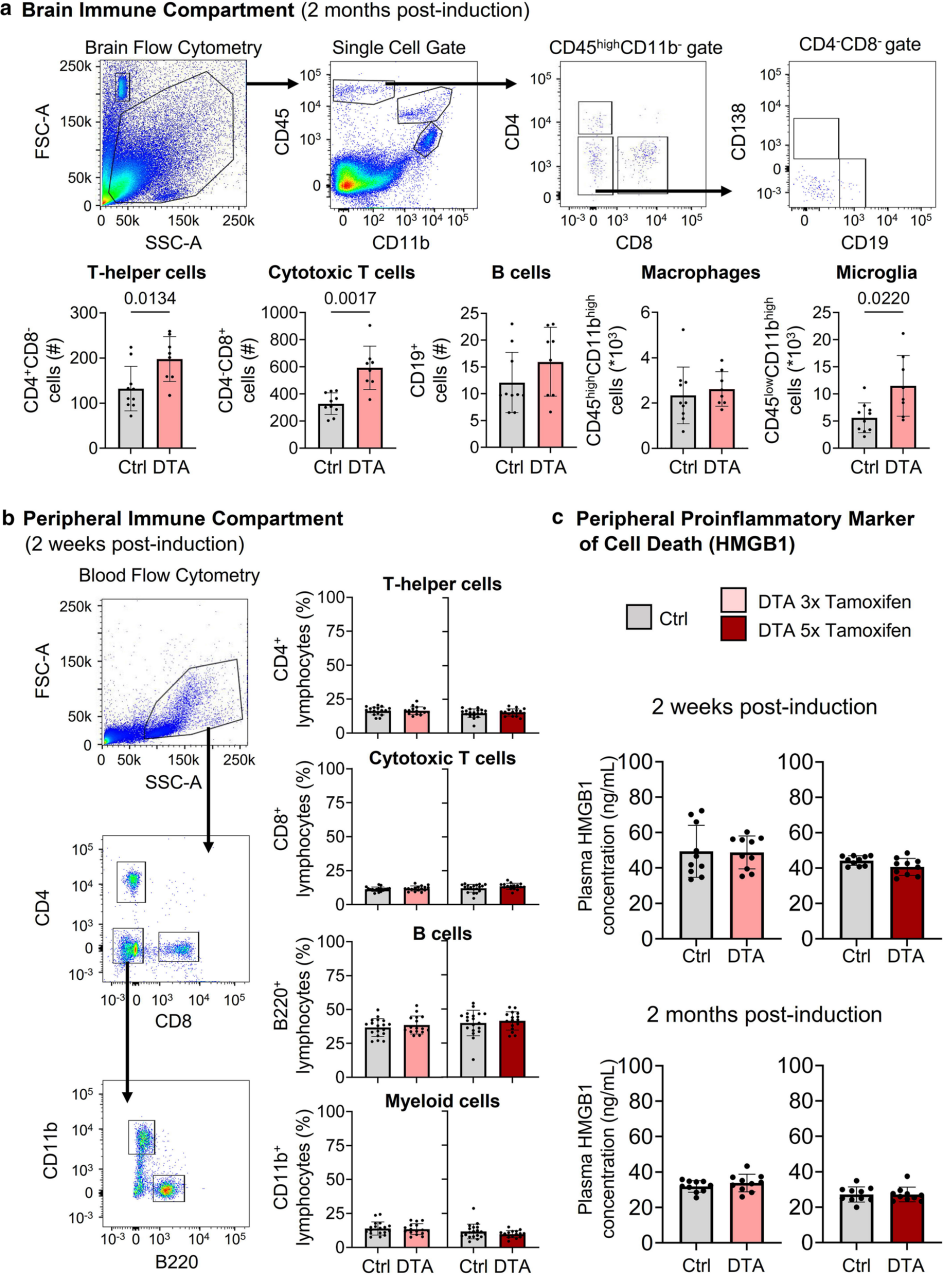
Evaluation of the brain and peripheral immune compartments. **a** Flow cytometry of brain tissue from DTA and control mice 2 months after 3 × tamoxifen induction. Gating strategy is depicted in the top row. DTA mice showed stronger infiltration of T-helper cells (CD11b^−^, CD45^high^, CD8^−^, CD4^+^), cytotoxic T cells (CD11b^−^, CD45^high^, CD4^−^, CD8^+^), and increased microglia numbers (CD11b^high^, CD45^low^). B cell numbers (CD11b^−^, CD45^high^, CD8^−^, CD4^−^, CD19^+^) were low in brain tissue of both groups. Macrophage numbers (CD11b^high^, CD45^high^) were not different. Data from 8–10 mice/group. **b** Flow cytometry of lymphocytes derived from peripheral blood 2 weeks after 3 × and 5 × tamoxifen induction. Gating strategy in left panel. DTA and control mice showed comparable and physiological numbers of major lymphocyte subsets (right panel). Data from 16–19 mice/group. **c** HMGB1 ELISA at 2 weeks and 2 months after 3 × or 5 × tamoxifen induction shows no difference in plasma concentrations between groups. Data from 10 mice/group. Data displayed as mean ± SD; 2-tailed unpaired Welch’s corrected *t*-tests or Mann–Whitney U-tests

Brain flow cytometry after 2 months of DTA induction (3 × tamoxifen) confirmed not only the histologically quantified microgliosis but also revealed persistent increases in T cells, in particular CD8^+^ cytotoxic T cells, shown very recently to drive axon degeneration in the normal aging mouse CNS and contribute to age-related cognitive and motor decline [16]. B cell numbers were low and like macrophages unaltered (Fig. 6a).

In contrast, blood flow cytometry at 2 weeks after DTA induction (in 3 × and 5 × tamoxifen mice) and upon considerable acute brain inflammation did not show any immune cell alterations in the periphery (Fig. 6b). This complete lack of peripheral changes reflects in our mouse model the enormous and well-known clinical problems of diagnosing even substantial brain inflammation in vivo, often leading to wrong assumptions regarding suddenly occuring behavioral abnormalities in neuropsychiatric practice.

In an approach to identify a possible peripheral diagnostic marker, we came across an alarmin, namely high-mobility group box 1 (HMGB1), a key molecule of host defense systems that can be released under acute and chronic pathological conditions. HMGB1 is a non-histone nuclear protein with dual functions depending on localization. Within cells, it is confined primarily to the nucleus where it binds DNA and plays a role in transcriptional regulation. However, extracellular HMGB1 serves as proinflammatory cytokine. Elevated HMGB1 levels in serum and CSF have been reported in neurological infection, indicating that excessive host inflammation may be relevant to the pathogenesis of encephalitis. Beyond infections, HMGB1 has pathogenic roles during trauma and sterile inflammation, such as systemic inflammatory response syndrome [14, 15, 30, 36, 44]. Thus, we followed HMGB1 plasma levels at 14 days and 2 months after DTA induction in both 3 × and 5 × tamoxifen mice. Surprisingly, there were no differences between groups at any time point or induction scheme (Fig. 6c).

Again, it is fascinating, but certainly also alarming for clinicians, that extensive inflammatory degeneration in the brain, including BBB breakdown, can occur without indication in the blood. Since this clear result is of major relevance also for human neurodegenerative disease, it will hopefully stimulate the development of even more sophisticated brain imaging paradigms, sensitive enough for in vivo diagnosis of ongoing brain inflammation.

### Modeling aspects of viral gray matter encephalitis by sterile pyramidal neuronal death: Synopsis and outlook

With the present longitudinal study, we aimed at defining downstream consequences of sudden pyramidal neuronal loss as it characterizes many viral infections, including reactive neuroinflammatory and subsequent neurodegenerative processes. Employing DTA mice, we present an animal model that allows sterile and targeted induction of cell death, thereby excluding the numerous parallel cellular and molecular events that characterize infections from the beginning and make delineation of causes and consequences often impossible. We provide not only the predictable histological evidence of a dose-dependent mild to moderate encephalitis together with the expected behavioral consequences of hippocampal and (pre)frontal damage. We also deliver clearcut and easily quantifiable novel results on disturbed social cognition with pathological thermoreaction/autonomic dysfunction as well as multifaceted sophisticated MRI readouts as future endpoints of targeted therapeutic approaches.

## Supplementary Information

**Additional file 1.**

## Data Availability

All data are available upon request.
